# An Empirical Study on the Equity Performance of China's Health Insurance Companies During the COVID-19 Pandemic—Based on Cases of Dominant Listed Companies

**DOI:** 10.3389/fpubh.2021.663189

**Published:** 2021-05-10

**Authors:** Rendao Ye, Na An, Yichen Xie, Kun Luo, Ya Lin

**Affiliations:** ^1^School of Economics, Hangzhou Dianzi University, Hangzhou, China; ^2^Alibaba Business College, Hangzhou Normal University, Hangzhou, China

**Keywords:** COVID-19 pandemic, uncertain impact, stock price of the health insurance company, ARIMA model, BP neural network model, LSTM neural network model

## Abstract

The health insurance industry in China is undergoing great shocks and profound impacts induced by the worldwide COVID-19 pandemic. Taking for instance the three dominant listed companies, namely, China Life Insurance, Ping An Insurance, and Pacific Insurance, this paper investigates the equity performances of China's health insurance companies during the pandemic. We firstly construct a stock price forecasting methodology using the autoregressive integrated moving average, back propagation neural network, and long short-term memory (LSTM) neural network models. We then empirically study the stock price performances of the three listed companies and find out that the LSTM model does better than the other two based on the criteria of mean absolute error and mean square error. Finally, the above-mentioned models are used to predict the stock price performances of the three companies.

## Introduction

The COVID-19 pandemic is first and foremost inducing instability and uncertainty in the high-quality development of China's economy, not only impacting industries of transportation and tourism but also causing significant fluctuations in the financial market (1–4). As a barometer of the economy, stock prices reflect the current conditions and future trends in the development of industries. During the pandemic, China's stock market is on the whole decline, while the stock prices of the health insurance sector continue to rise as market demand expands. It is of practical significance to study the stock price performances of China's health insurance companies during the pandemic and to provide insights into the impacts of the pandemic and trends in the development of this industry.

The impacts of the COVID-19 pandemic on the stock market have become a focus in recent research. Duan (5) explored the impacts of the pandemic on the stock returns of listed companies in China's pharmaceutical industry using the event analysis method. The empirical results show that the pandemic is having a significantly positive short-term impact on the stock returns in this industry. Based on data of 3,550 listed companies in China in the early stage of the pandemic, Wang et al. (6) studied the impacts of new confirmed cases in the place of company registration on the fluctuations of stock prices. Using the panel vector autoregression model, individual fixed effects model, and dynamic econometric model, they found a U-shaped relationship between daily stock prices and numbers of new confirmed cases. Similarly, Xia and Hu (7) used the Fama-French three-factor model and panel regression to analyze the performances of 223 pharmaceutical stocks during the pandemic in Shanghai and Shenzhen 300 index. Sun et al. (8) found out, in their case studies, about a stronger positive correlation between investor sentiment and stock returns during the pandemic than in previous periods. Mazur et al. (9) explored the stock market performances in the United States during the pandemic and found that the stock prices in natural gas, food, healthcare, and software industries showed an upward trend, while the stock prices in oil, real estate, entertainment, and hotel industries showed the opposite. Heyden and Heyden (10) studied the short-term reaction in stock markets of the United States and Europe at the early stage of the pandemic. Their results showed that fiscal measures had a negative impact on stock returns, while monetary policy had a stabilizing effect on the market.

Stock price fluctuations during the pandemic directly affect the stability of the financial market and healthy development of national economy. Thus, the prediction of stock price performances becomes a hot topic in recent research. The most often used methods include the autoregressive integrated moving average (ARIMA), back propagation (BP) neural network, and long short-term memory (LSTM) neural network models. Bai (11) and Shi et al. (12) used the ARIMA method to model the stock prices of the Shanghai composite index, established an improved model in their short-term prediction, and then proved the effectiveness of both models. Chen (13) constructed the ARIMA and BP neural network models to predict the stock prices of two famous companies in the IT industry of China, namely, Baidu and Alibaba. Both models are found to have ideal prediction accuracy and short-term prediction effect. Some research specifically aim to tackle the redundant problem of the experimental samples. For example, Cai and Chen (14) and Huo et al. (15), respectively, proposed the stock price prediction models of the principal component analysis–BP neural network and LM–BP algorithm and verified their high accuracy in short-term prediction. With a view to improve prediction accuracy, Peng et al. (16) constructed the LSTM neural network model of different layers for stock price prediction and found out about the appropriate numbers of LSTM layers and hidden neurons. Furthermore, Song et al. (17) proposed a LSTM neural network model based on particle swarm optimization, which matched the characteristics of stock prices with network topology so as to improve prediction accuracy. However, research and studies have mostly focused on the stock price performances in industries of real estate, Internet, medicine, and some others. With the COVID-19 pandemic ongoing and health insurance becoming a most important foundation of people's livelihood, the prediction of stock prices in this industry is of great significance for analyzing its prospects. Seeing this, this paper constructs a stock price prediction method using the ARIMA, BP neural network, and LSTM neural network models to study the impacts of the pandemic on China's health insurance industry from the perspective of stock price fluctuations.

This paper is organized as follows: section Trend Analysis of Stock Closing Prices makes a descriptive statistical analysis of the stock prices of the three dominant health insurance listed companies during trading days from 2015 to 2020. In section Methodology, we introduce three prediction models, namely, the ARIMA, BP neural network, and LSTM neural network models. Those models are applied to the stock price predictions of the three companies in section Stock Price Prediction, and a comparative analysis of the empirical results using various models is also given. Section Conclusion and Prospect gives the conclusion of this paper.

## Trend Analysis of Stock Closing Prices

### Variables

This paper observes data of stock closing prices of the three dominant health insurance listed companies, namely, China Life Insurance, Ping An Insurance, and Pacific Insurance, in 1,461 consecutive trading days from January 1, 2015 to December 31, 2020. Lagrange's interpolation is used to solve the problem of missing data of Pacific Insurance for 16 trading days (from January 15 to 22, 2016 and February 3 to 16, 2017).

### Descriptive Analysis

**Figure 1:**
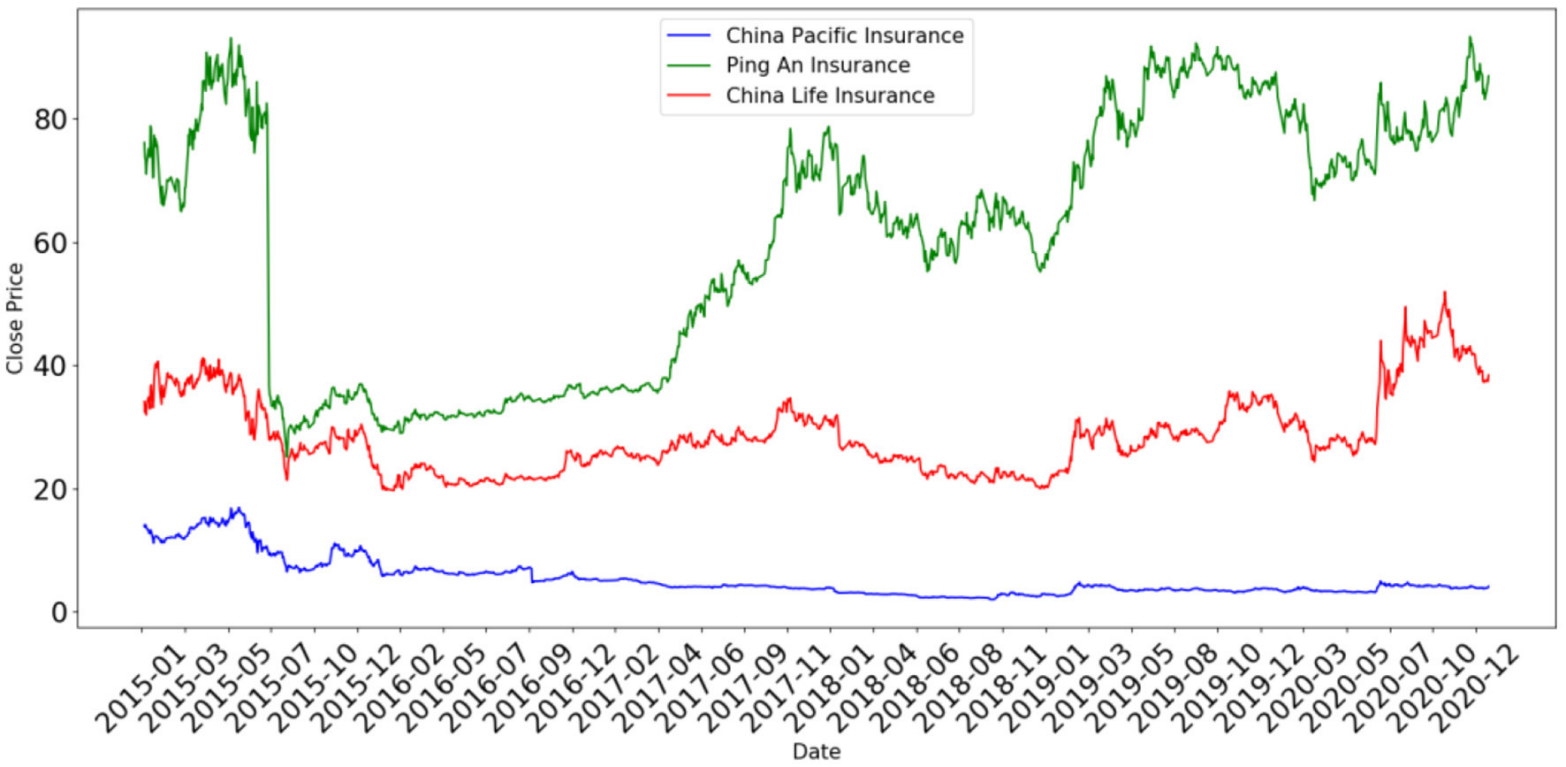
Time series charts of the stock closing prices of the three health insurance companies. The data are from NetEase Finance (http://quotes.money.163.com/ stock).

Figure 1 gives the time series charts of the stock closing prices of the three companies, namely, China Life Insurance, Ping An Insurance, and Pacific Insurance from 2015 to 2020, respectively.

Figure 1 shows that the stock price fluctuations of China Life Insurance and Ping An Insurance were roughly the same during this period. As a matter of fact, the stock prices of these two companies seemed to have been fluctuating with the market. Soon after the outbreak of the pandemic, the stock prices of both companies fell sharply with the entire market in quarter 1 of 2020. Then, from quarter 2 with the advancement of China's pandemic prevention and control, economic growth turned positive, and the stock market gradually recovered. The stock prices of both companies even rose to levels higher than before the outbreak. For example, the stock closing price of China Life Insurance reached to a peak of 51.96 RMB on October 21, 2020, higher by 49.01% than that on December 31, 2019. Even after a gradual fall and hitting a bottom of 37.25 RMB on December 24, 2020, the stock price was still higher than before the pandemic. Similar stories could be observed in the case of Ping An Insurance. Furthermore, Figure 1 indicates something different for Pacific Insurance. Before the outbreak of the pandemic, its stock prices had been undergoing an overall decline over years since reaching a peak in June 2015. However, the stock price still showed a slight increase in April 2020.

Table 1 further depicts the changes in stock prices of the above-mentioned three companies before and after the pandemic, where MAX and MIN, respectively, denote the maximum and minimum of the stock closing prices, and CV denotes the coefficient of variation.

**Table 1:** Descriptive statistics of the stock closing prices of three health insurance companies.

**Company**	**Period**	**MAX**	**MIN**	**Mean**	**CV**
China Life Insurance	Before the outbreak	41.19	19.66	27.08	0.18
	After the outbreak	51.96	24.33	35.65	0.21
Ping An Insurance	Before the outbreak	93.17	25.11	57.51	0.36
	After the outbreak	93.38	66.76	78.67	0.07
Pacific Insurance	Before the outbreak	16.92	1.97	5.62	0.60
	After the outbreak	4.92	3.10	3.76	0.11

*Since December 31, 2019, the Health Commission of Wuhan Municipality started to release the pandemic information. Take this day as a symbolic date of pandemic outbreak with an outburst of new confirmed cases nationwide*.

From Table 1, the MAX, MIN, and mean values of stock prices of China Life Insurance and Ping An Insurance after the outbreak of the pandemic were all higher than before, indicating that both companies were impacted at least not negatively. As for Pacific Insurance, since its stock prices had been continuing to decline for years, it would not be appropriate to use the MAX and mean values to illustrate the changes in stock prices before and after the pandemic, and thus the MIN values were observed instead. The results show that the MIN value after the outbreak was significantly higher than before. It appears that the stock prices of all three companies were boosted, only to different extents. In addition, the CV indexes show a slight increase in the deviation of stock prices for China Life Insurance, with significant decreases for the other two companies, indicating that their stock prices were fluctuating less intensely than before. In summary, although shocked by the outbreak of the pandemic in the short term, the stock prices of the three dominant health insurance companies recovered quickly with the gradual advancement of China's pandemic prevention and control and the resumption of work and production. The stock prices showed an obvious upward trend and, in some cases, even rose above previous levels and fluctuate less fiercely.

## Methodology

To study the impacts of the pandemic on the stock price performances, we use the three models of ARIMA, BP neural network, and LSTM neural network to construct a stock price prediction methodology. The ARIMA model uses the weighted modeling of the historical value, current value, and lagged stochastic disturbance term to explain and predict the trend of time series. The BP and LSTM neural network models use activation function and network layer extension to enhance the processing ability of nonlinear data and therefore are widely used in stock price prediction.

### ARIMA Model

ARIMA (*p, d, q*) is a differential autoregressive (AR) moving average (MA) model, where *p, q*, and *d* denote the orders of the AR process, MA process, and difference required to change the original time series into stationary time series, respectively. Since the ARIMA model is a combination of differential operation and ARMA model, the model can be expressed as follows (18):

$$\begin{array}{l} \{\begin{array}{l} \Phi \left(B\right)\nabla^{d}x_{t}=\theta \left(B\right)\varepsilon_{t}\\ E\left(\varepsilon_{t}\right)=0,Var\left(\varepsilon_{t}\right)=\sigma_{\varepsilon }^{2},E\left(\varepsilon_{t}\varepsilon_{s}\right)=0,s\neq t\\ E\left(\varepsilon_{t}\varepsilon_{s}\right)=0,\forall s<t, \end{array} \end{array}$$

where B is the delay operator, ∇^*d*^ = (1 − *B*)^*d*^, $\Phi \left(B\right)=1-\varphi_{1}B-\cdots -\varphi_{p}B^{p}$ is the AR polynomial of the ARMA model, and $\theta \left(B\right)=1-\theta_{1}B-\cdots -\theta_{q}B^{q}$ is the MA polynomial of the ARMA model. ε_*t*_ denotes the random error, and φ_*i*_ and θ_*j*_ are the AR coefficient and MA coefficient of the ARMA model, respectively. The flow chart is described as follows.

**Figure 2:**
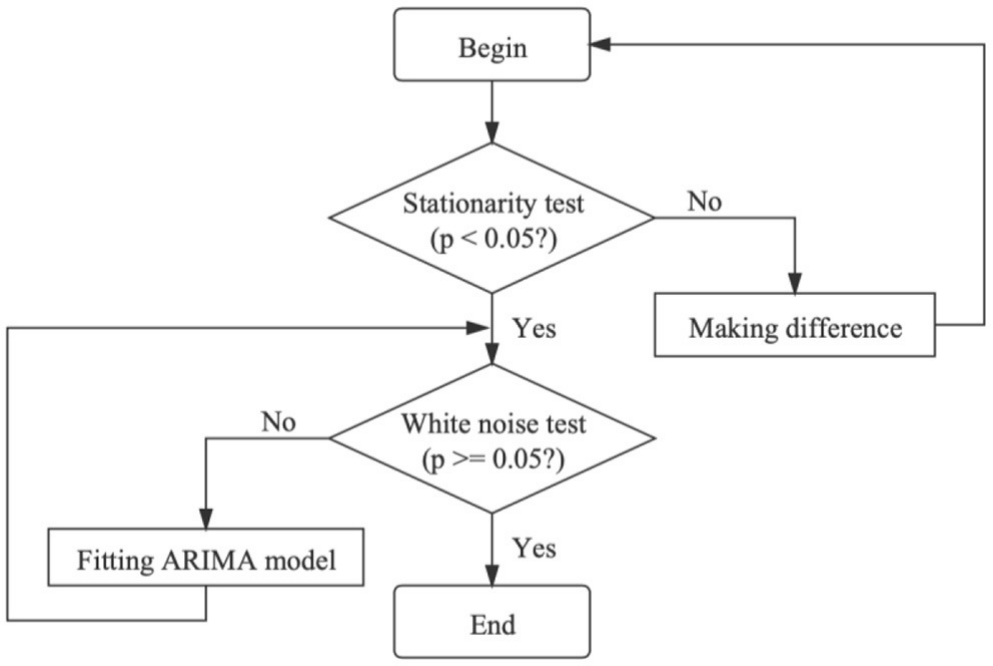
Flow chart of the autoregressive integrated moving average model [from Li et al. (18)].

As shown in Figure 2, we firstly perform the stationarity test for the original time series and then make a difference for non-stationary data. Secondly, the parameters of stabilized data are estimated by the auto.arima function and BIC heat map in R software, and the white noise test is used to judge whether the residual is a white noise sequence. Finally, the data are analyzed and predicted by the significant model.

### BP Neural Network Model

**Figure 3:**
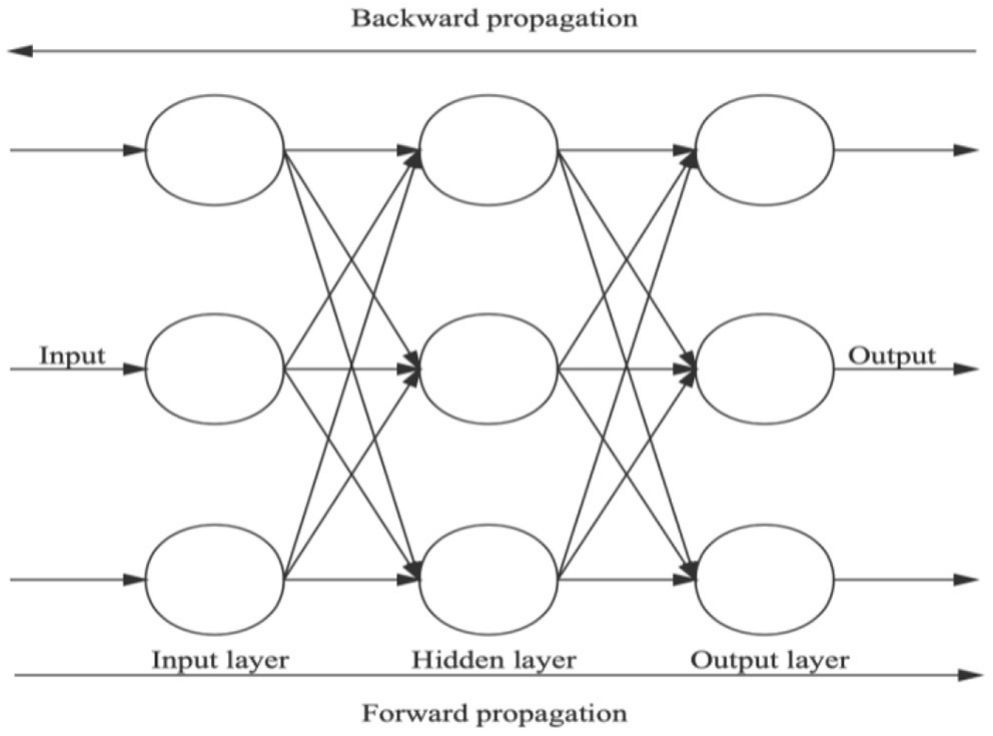
Three-layer back propagation neural network topology [from Chen et al. (19)].

The BP neural network model is the activation function model of linear weight (19). The learning process is divided into two parts, including forward propagation process and backward propagation process, as shown in Figure 3.

Forward propagation refers to the process in which neurons in the input layer receive external information and then pass it to the hidden layer. Through the control and conversion of the hidden layer, the output layer finally outputs the calculation results. The backward propagation process is aimed at the error, using the gradient descent method to adjust the continuous parameters for each layer, and the propagation direction is transferred from the output layer to the input layer. Through forward propagation and backward propagation processes, the connection parameters among layers are gradually optimized. For the BP neural network stock price prediction model, the network structure is set as follows:

1. The number of network layers: Since the three-layer BP neural network model can fit any non-linear function with any accuracy and the increase in the number of hidden layers does not significantly improve the generalization ability and prediction accuracy of the network, a three-layer BP neural network structure including only one hidden layer is used for learning.
2. Number of neurons in each layer: Select the opening price, highest price, lowest price, trading volume, and closing price of *N* consecutive trading days as the network input. The predicted result is the closing price of the next trading day, so the numbers of neurons in the input layer and output layer are 5*N* and 1, respectively. The number of neurons in the hidden layer of the BP neural network is 3 by the method of trial and error.
3. Activation function: Since the sigmoid function can compress the input value with a large range of changes to the interval (0, 1), the processing capacity of the network is greatly improved. Therefore, this paper uses the sigmoid function in the BP neural network model.
4. Training method and optimizer selection: The goal of this paper is to predict the future closing price of stocks; thus, MSE is selected as the loss function. Due to the characteristics of fast convergence speed and good learning effect, the Adam optimizer is used for optimization training.

### LSTM Neural Network Model

Based on the BP neural network model, the LSTM neural network model is obtained by solving the long sequence dependency problem of time series data. The network structure of the model adopts a control gate mechanism, which is composed of memory cells, input gates, output gates, and forget gates (16). The calculation steps for each control gate are as follows:

Let *W*_*i*_, *W*_*c*_, *W*_*f*_, and *W*_0_ denote the weights and *b*_*i*_, *b*_*c*_, *b*_*f*_, and *b*_*o*_ denote the biases; *X*_*t*_ denotes input at time *t*, and *h*_*t*−1_ denotes memory state information at time *t* − 1. Firstly, calculate the value of the input gate *i*_*t*_ at time *t* and the candidate state value ${\tilde{C}}_{t}$ of the input cell:

$$\begin{array}{l} i_{t}=\delta \left(W_{i}*\left(X_{t},h_{t-1}\right)+b_{i}\right),{\tilde{C}}_{t}=\tanh\left(W_{c}*\left(X_{t},h_{t-1}\right)+b_{c}\right), \end{array}$$

where δ represents the activation function. Secondly, calculate the activation value *f*_*t*_ of the forget gate at time *t*:

$$\begin{array}{l} f_{t}=\delta \left(W_{f}*\left(X_{t},h_{t-1}\right)+b_{f}\right). \end{array}$$

Furthermore, the cell state update value *C*_*t*_ at time *t* can be obtained:

$$\begin{array}{l} C_{t}=i_{t}*{\tilde{C}}_{t}+f_{t}*C_{t-1}. \end{array}$$

Finally, calculate the value of the output gate *O*_*t*_:

$$\begin{array}{l} O_{t}=\delta \left(W_{o}*\left(X_{t},h_{t-1}\right)+b_{o}\right). \end{array}$$

Through the steps given above, the LSTM neural network structure can effectively use the input to make itself have a long-term memory function. Furthermore, the LSTM neural network structure is set as follows:

1. The number of network layers: The LSTM neural network model uses a two-layer hidden layer network. Then, the problem of overfitting is solved by the dropout function.
2. The number of neurons in each layer: Set the number of neurons in the input layer and output layer to five and one, respectively. Furthermore, the number of neurons in the first hidden layer is 60, and the number of neurons in the second hidden layer is 120 by the method of trial and error.
3. Time step: For selecting the network input sequence, we take the stock data of *N* consecutive trading days as a set of input, and the purpose of learning is to obtain the closing price of the stock on the *N* + 1 trading day. In this paper, the step size *N* is set to 5 and 10, respectively, and comparative analysis is performed.
4. Activation function: Replace the sigmoid function with the ReLU function to solve the vanishing gradient problem. When the input of the activation function is negative, the output is 0, and the neuron will not be activated. Only some neurons are activated at the same time, which makes the network sparse and improves computational efficiency.

## Stock Price Prediction

### Prediction With ARIMA Model

#### Stationarity Test

**Figure 4:**
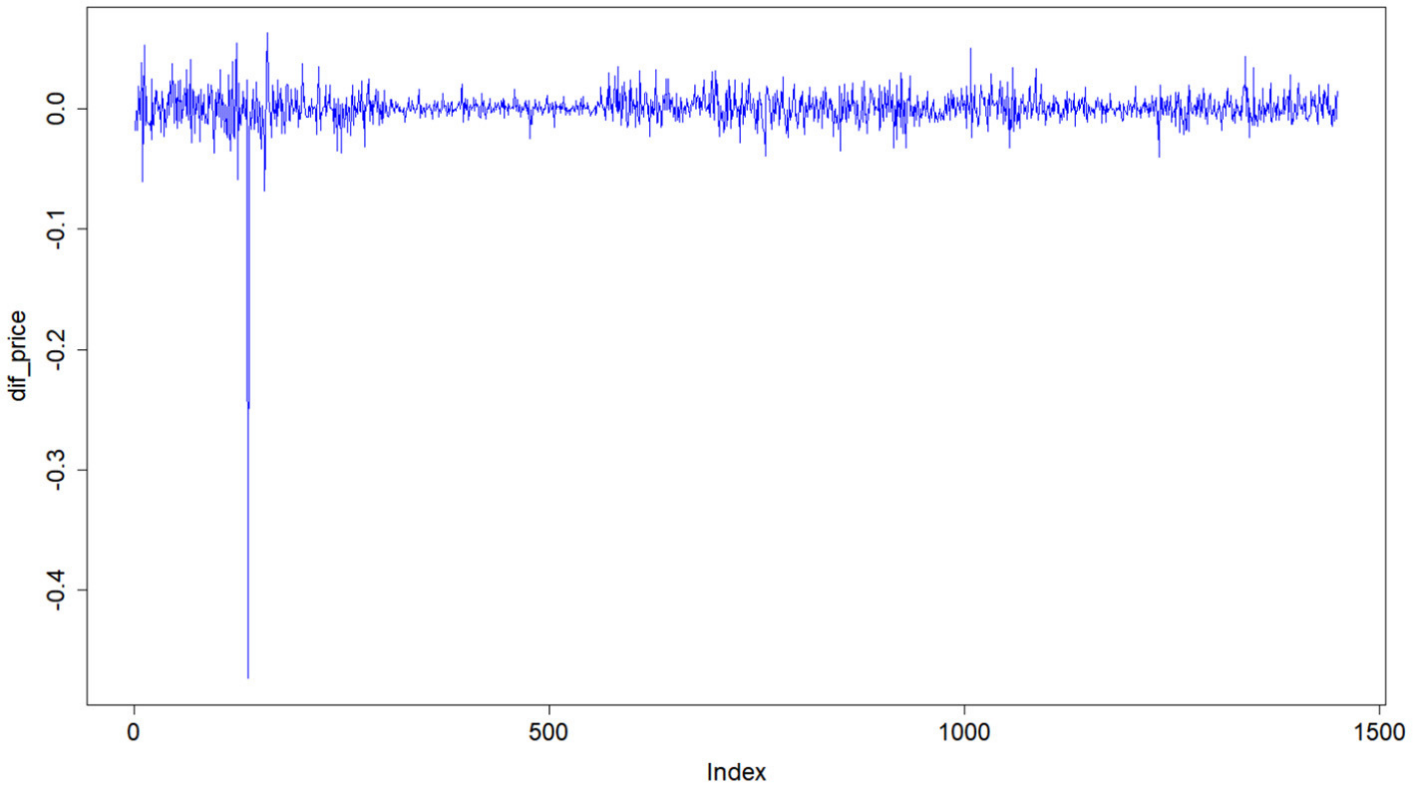
Time series diagram after the first order difference of Ping An Insurance.

To improve the accuracy of stock price prediction, we carry out a Box–Cox transformation for the original data of Ping An Insurance. Since the stock prices of Ping An Insurance are non-stationary, as shown in Figure 1, the data is stabilized by the first-order difference (see Figure 4). The time series of the stock prices after the first-order difference fluctuates around the constant 0, and the *p*-value of the unit root test is <0.05, that is, the data are stabilized after the first-order difference. Similarly, the data of China Life Insurance and Pacific Insurance are stabilized after the first- and second-order differences, respectively.

#### Model Fitting

**Figure 5:**
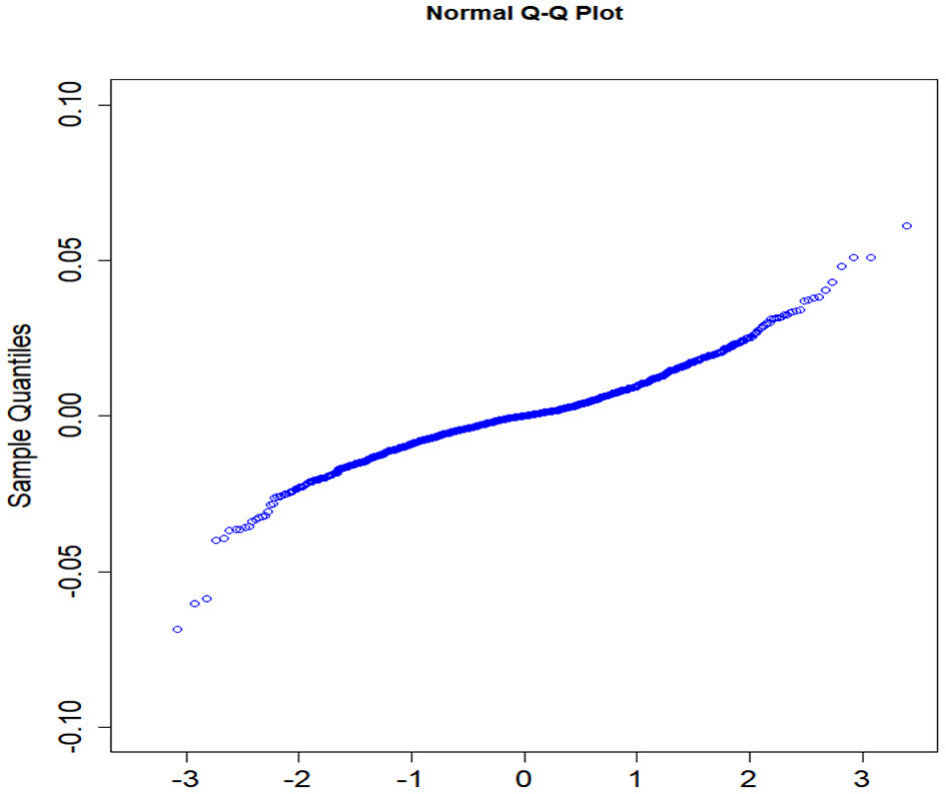
Q-Q plot of Ping An Insurance.

The parameters *p* and *q* are determined by the auto.arima function in R software, and the fitting model of the stock price of Ping An Insurance is ARIMA (1, 1, 1). Then, *Q*–*Q* plot is used to conduct the normality test for the residual (see Figure 5). The *x*-coordinate and *y*-coordinate denote the quantiles of normal distribution and sample, respectively. From Figure 5, the stock price residual of Ping An Insurance approximately follows a normal distribution. Furthermore, the *p*-value of the white noise test is higher than 0.05, that is, the residual is a white noise sequence, so the fitting model is significantly effective. Similarly, it can be proved that the fitting model of the stock prices for China Life Insurance is ARIMA (1, 1, 1) and that for Pacific Insurance is ARIMA (1, 2, 1).

### Prediction Effect of the ARIMA Model

In this section, the ARIMA model is constructed to predict the stock prices of the three health insurance companies. Sample data are taken from the stock closing prices of the above-mentioned companies in trading days from December 18 to 31, 2020. Table 2 gives the specific prediction results and their errors. The error range between the predicted values and actual values of the three companies is 0–6.96%, showing a high prediction accuracy of the model and an accurate reflection of fluctuations in stock prices. Furthermore, the MAE and MSE of the stock prices of the three companies are calculated (see Table 3). The MAE of the ARIMA model ranges from 0.0880 to 3.5670, and the MSE ranges from 0.0120 to 14.7188. Based on these criteria, the ARIMA model is considered to have presented a good fitting effect.

**Table 2:** Prediction results and errors of stock prices of three health insurance companies.

**Date**	**Company**	**Actual value**	**Predicted value**	**Prediction error**
2020/12/18	China Life Insurance	38.91	39.84	2.39%
	Ping An Insurance	87.79	88.99	1.37%
	Pacific Insurance	3.83	3.87	1.04%
2020/12/21	China Life Insurance	39.09	39.83	1.89%
	Ping An Insurance	87.05	88.98	2.22%
	Pacific Insurance	3.87	3.87	0.00%
2020/12/22	China Life Insurance	37.38	39.83	6.55%
	Ping An Insurance	84.15	88.99	5.75%
	Pacific Insurance	3.73	3.87	3.75%
2020/12/23	China Life Insurance	37.40	39.83	6.50%
	Ping An Insurance	84.85	88.99	4.88%
	Pacific Insurance	3.76	3.87	2.93%
2020/12/24	China Life Insurance	37.25	39.83	6.93%
	Ping An Insurance	84.20	88.99	5.69%
	Pacific Insurance	3.72	3.86	3.76%
2020/12/25	China Life Insurance	37.52	39.83	6.16%
	Ping An Insurance	83.20	88.99	6.96%
	Pacific Insurance	3.80	3.86	1.58%
2020/12/28	China Life Insurance	37.40	39.83	6.50%
	Ping An Insurance	84.62	88.99	5.16%
	Pacific Insurance	3.80	3.86	1.58%
2020/12/29	China Life Insurance	37.71	39.83	5.62%
	Ping An Insurance	85.50	88.99	4.08%
	Pacific Insurance	3.84	3.86	0.52%
2020/12/30	China Life Insurance	37.38	39.83	6.55%
	Ping An Insurance	85.88	88.99	3.62%
	Pacific Insurance	3.94	3.86	2.03%
2020/12/31	China Life Insurance	38.39	39.83	3.75%
	Ping An Insurance	86.98	88.99	2.31%
	Pacific Insurance	4.08	3.85	5.64%

**Table 3:** Prediction effect of the autoregressive integrated moving average (ARIMA) model.

**Model**	**Mean absolute error**	**Mean square error**
ARIMA (1,1,1) (China Life Insurance)	3.5670	14.7188
ARIMA (1,1,1) (Ping An Insurance)	1.9880	4.3788
ARIMA (1,2,1) (Pacific Insurance)	0.0880	0.0120

### Prediction With BP and LSTM Neural Network Models

#### Data Normalization

The degrees of gradient descent for each parameter are proportional to the magnitude in training the neural network model. However, the parameters in this paper are different in magnitude, so it is necessary to normalize the original data and map the data to the interval [0, 1] by the following formula:

$$\begin{array}{l} x_{n}=\frac{x-x_{\min}}{x_{\max}-x_{\min}}, \end{array}$$

where *x*_*n*_ and *x* represent the normalized data and original data, respectively, and *x*_min_ and *x*_max_ represent the minimum and maximum of the original data, respectively. In particular, to facilitate the subsequent comparative analysis, we make the reverse normalization of the results and restore the data to the original magnitude.

### Prediction Effects of BP and LSTM Neural Network Models

In this section, Keras in Python is used to construct the BP and LSTM neural network models. The 5- and 10-day stock data are used as the input samples to build the prediction models. Let RS, PA, and TPY represent the three companies of China Life Insurance, Ping An Insurance, and Pacific Insurance, respectively. Besides this, Y6, D5, and D10 represent the span of the data set of 6 years and the samples of five consecutive trading days and 10 consecutive trading days, respectively. The prediction effects of the BP and LSTM neural network models are measured by the MAE, MSE, and discriminant coefficient *R*^2^. The results are given in Table 4.

**Table 4:** Prediction effects of back propagation (BP) and long short-term memory (LSTM) neural network models.

**Model structure**	**Mean absolute error**		**Mean square error**		***R*^2^**	
	**BP**	**LSTM**	**BP**	**LSTM**	**BP**	**LSTM**
RSY6D5	1.4255	1.2417	5.6332	4.7662	0.8770	0.8960
PAY6D5	1.8684	1.6298	5.4513	4.1271	0.9150	0.9357
TPYY6D5	0.1544	0.1086	0.0325	0.0294	0.8261	0.8429
RSY6D10	2.1694	0.9474	10.2784	1.9913	0.7756	0.9565
PAY6D10	3.4425	1.5166	18.1472	3.7957	0.7171	0.9408
TPYY6D10	0.1742	0.0986	0.0754	0.0220	0.5963	0.8822

Under the structures of Y6 and D5, we further analyze the prediction effects for the three companies with two neural network models. Under the RSY6D5 structure, compared with the BP neural network model, the MAE and MSE of the LSTM neural network model decreased by 12.89 and 15.39% respectively, while *R*^2^ increased by 2.17%. The MAE and MSE of the LSTM neural network model under the PAY6D5 structure decreased by 12.77 and 24.29%, respectively, and *R*^2^ increased by 2.26%. The MAE and MSE of the LSTM neural network model under the TPYY6D5 structure are reduced by 29.66 and 9.54%, respectively, and *R*^2^ increased by 2.03%, that is, for all the three observed companies, the prediction effect of the LSTM neural network model is better than that of the BP neural network model, and it still holds under the structures of Y6 and D10.

**Figure 6:**
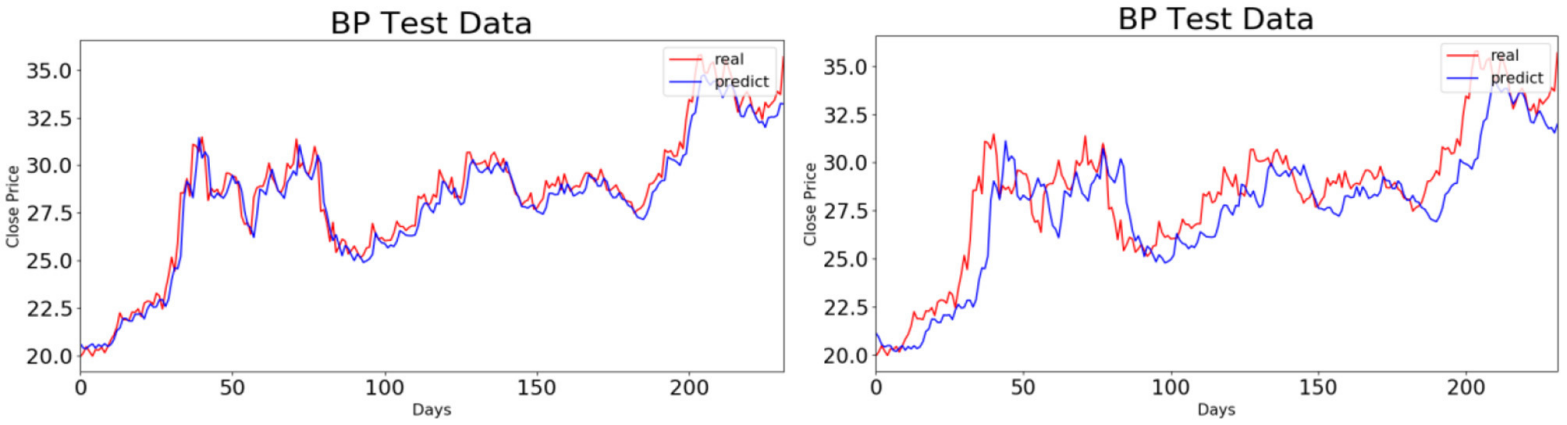
Fitting effect diagrams of back propagation neural network model in two test sets.

**Figure 7:**
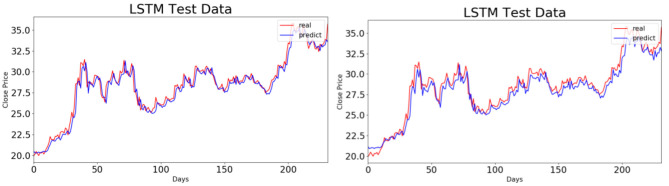
Fitting effect diagrams of long short-term memory neural network model in two test sets. The diagrams on the left and right represent RSY6D5 and RSY6D10, respectively.

To compare the BP and LSTM neural network models graphically, we take China Life Insurance, for instance, to give the fitting effect diagram of the above-mentioned two models in the test set. The results are shown in Figures 6, 7. When the BP neural network model is used for prediction, the prediction model with a time step of five trading days has a higher accuracy than the one with a time step of 10 trading days. When the LSTM neural network model is used, however, there is no significant difference in the prediction effects under the above-mentioned two kinds of time steps. Combined with Table 4, it is obvious that the prediction effect of the LSTM neural network model improves with the increase in time steps.

In summary, for the three health insurance companies that we observed, the stock price prediction effect of the LSTM neural network model is better than those of the ARIMA and BP neural network models under the MAE and MSE criteria. Moreover, the prediction effect of the LSTM neural network model improves with the increase in time steps, while the prediction effect of the BP neural network model shows the opposite. It is also found that the LSTM neural network model could deal with the problem of longer sequences and excavate the information of long-term dependence. Therefore, we consider it more appropriate to use the LSTM neural network model in stock price prediction.

Based on the above-mentioned analysis, we use the ARIMA model, BP neural network model with a time step of 5, and LSTM neural network model with a time step of 10 to predict the stock closing prices of China Life Insurance, Ping An Insurance, and Pacific Insurance on January 4, 2021. The predicted results are shown in Table 5.

**Table 5:** Predicted stock closing prices on January 4, 2021.

**Company**	**Model**		
	**Autoregressive integrated moving average (ARIMA)**	**Back propagation (BP)**	**Long short-term memory**
China Life Insurance	39.83	36.62	38.10
Ping An Insurance	89.23	83.98	85.84
Pacific Insurance	3.85	3.90	3.92

*ARIMA and BP models with a time step of 5, LSTM model with a time step of 10*.

## Conclusion and Prospect

This paper mainly studies the equity performance of China's health insurance companies during the COVID-19 pandemic, aiming to analyze the impacts of the pandemic on the stock prices of dominant companies and establish more accurate stock price prediction models. Clearly, the stock prices of China Life Insurance, Ping An Insurance, and Pacific Insurance fell overall in the first quarter of 2020. With the advancement of China's pandemic prevention and control as well as the resumption of work and production, the stock prices quickly recovered and showed an overall rising trend. In spite of slight fluctuations, the stock prices generally rose above levels before the pandemic.

Then, three models of the ARIMA, BP, and LSTM neural network are used to predict the stock prices of the three health insurance companies in China. Firstly, for the ARIMA model, the parameters of the stabilized data are estimated by the auto.arima function and BIC heat map in R software, and a significant model is obtained by the white noise test. By calculation, the value ranges of MAE and MSE are 0.0880–3.5670 and 0.0120–14.7188, respectively, that is, the ARIMA model performs well in the fitting. Secondly, based on the normalized stock price data of the three companies, BP and LSTM neural network models are trained by Python and applied to the test set data. The LSTM neural network model is more accurate in stock price prediction than the BP neural network model under the criteria of MAE, MSE, and *R*^2^. In conclusion, the prediction effect of the LSTM neural network model is better than those of the ARIMA and BP neural network models under the criteria of MAE and MSE. Furthermore, since the LSTM neural network model can deal with longer time series problems, this paper recommends this model for predicting the stock prices of China's health insurance listed companies.

In this paper, we use three models to analyze the stock price trends of the three health insurance companies during the pandemic. The above-mentioned research results can be applied to the whole health insurance industry by expanding the sample scope and also provide references for stock price prediction in other industries such as the Internet and real estate. We will further our research in several aspects, including discussing the influence of time steps on the LSTM neural network model, in the future. This research will help to improve the fitting effect and prediction accuracy of the LSTM neural network model.

## Data Availability Statement

The original contributions presented in the study are included in the article/supplementary material, further inquiries can be directed to the corresponding author/s.

## Author Contributions

All authors listed have made a substantial, direct and intellectual contribution to the work, and approved it for publication.

## Conflict of Interest

The authors declare that the research was conducted in the absence of any commercial or financial relationships that could be construed as a potential conflict of interest.
